# Cysteine Pegylation of a Mutant L-asparaginase Affords Enhanced Activity and Thermostability. A Comparative Study Against N-terminal Conjugation

**DOI:** 10.1007/s12010-025-05545-1

**Published:** 2026-02-07

**Authors:** Rafael B. Ferraro, Guilherme R. Benevides, Jheniffer Rabelo, Flaviana da Silva Chaves, Grace Veronica Ruiz-Lara, Gustavo Carretero, Gisele Monteiro, Adalberto Pessoa-Junior, Attilio Converti, Steven Lynham, Paul F. Long, Carlota O. Rangel-Yagui

**Affiliations:** 1 Department of Biochemical Pharmaceutical Technology, School of Pharmaceutical Sciences, University of São Paulo, São Paulo, Brazil; 2 Institute of Pharmaceutical Science, Faculty of Life Sciences & Medicine, King’s College London, London, UK; 3 Institute of Chemistry, University of São Paulo, São Paulo, Brazil; 4 University of Genoa, Genoa, Italy; 5 Proteomics Core Facility, The James Black Center, King’s College London, London, UK

**Keywords:** L-Asparaginase, Pegylation, Enzymatic activity, Enzyme stability, Thermostability, Thermodynamics

## Abstract

L-asparaginase (ASNase) is one of the most clinically relevant biopharmaceuticals but proteolysis impairs the enzyme half-life. ASNase P40S/S206C was developed to overcome proteolysis and in addition pegylation can be used to improve half-life and thermostability. Here Cys206 and N-terminal residues were explored as pegylation sites for a potential new biobetter. Optimal mono-pegylation of Cys (Cys-PEG-ASNase) provided similar yields compared to N-terminal mono-pegylation (NT-PEG-ASNase) but at lower PEG concentration. Specific activity was higher for Cys-PEG-ASNase than NT-PEG-ASNase. The role of pegylation site in activity was confirmed by activation energy (*Ea*) and enthalpy variation (Δ*H*_*70ºC*_) of the ASNase-catalysed reaction. Pegylation in both sites increased enzyme thermostability and consequently shelf-life stability. The variation of Gibbs free-energy of enzyme thermo-inactivation (Δ*G*_*d*_) showed higher stabilization by Cys conjugation, while enthalpy (Δ*H*_*d*_) and entropy (Δ*S*_*d*_) evidenced an increase in aggregation upon thermo-inactivation, higher for Cys-PEG-ASNase.

**Supplementary Information:**

The online version contains supplementary material available at 10.1007/s12010-025-05545-1.

## Introduction

L-asparaginase (EC. 3. 5. 1. 1) (ASNase)is an important enzyme used to treat hematological malignancies, particularly Acute Lymphoblastic Leukemia since the 1970s and has led to a substantial improvement in cure rates, especially in children [1]. Lymphoblastic leukemic cells express very low levels of the enzyme asparagine synthetase,therefore, these cells are replete in asparagine and heavily depend upon extracellular sources of this amino acid. The therapeutic effect of ASNase is a complete and sustained depletion of serum asparagine, forcing cancerous cells into apoptosis [2]. Although ASNase is recognized as a highly efficient therapeutic agent, its use has several drawbacks such as immunogenicity [3] and degradation by the lysosomal proteases cathepsin B (CTSB) and asparaginyl endopeptidase (AEP) [4]. To address these problems, Rodrigues et al. [5] have developed a mutant ASNase P40S/S206C resistant to proteolytic cleavage by AEP and CTSB whilst retaining anti-cancer activity in murine models. Another alternative to reduce immunogenicity and increase ASNase half-life is pegylation.

Pegylation is defined as the covalent attachment of polyethylene glycol moieties to a protein and is one of the most widely employed techniques to develop superior biopharmaceuticals known as biobetters [6]. The selection of the pegylation site is fundamental since it can impact the enzyme activity, conformational stability, and aggregation, influencing not only storage and cold chain management but also clinical outcomes [7, 8]. Furthermore, the location and type of residue for conjugation also influence the optimal conjugation conditions [9]. Benefits of pegylation include an increase in hydrodynamic radius, leading to lower glomerular filtration and longer plasma half-life (*t*_1/2_), shielding effect against antibodies, increased stability in solution and enhanced thermostability [10]. Since marketed pegylated ASNase Oncaspar® is prepared by random pegylation at amino residues, it lacks proper control of the degree and site of pegylation, resulting in a polydisperse system and a lack of batch-to-batch reproducibility [11, 12]. Recently, site-specific N-terminal pegylation of ASNase and Crisantaspase (ASNase variant natively produced by *Erwinia Chrysantemi*) has been investigated by our group and a modified enzyme was found to preserve activity against leukemic cells in vitro [13, 14]. This modified enzyme was site-specifically pegylated at N-terminal residue using mPEG-N-Hydroxysuccinimide (mPEG-NHS).

In this work we pegylated the mutant *E. coli* ASNase P40S/S206C at two distinct residues, namely the N-terminal and Cys206, to investigate the effect of the pegylation site on the enzyme activity and stability. This is the first report, as far as we are aware, addressing differences regarding the conjugation sites on ASNase.

## Materials and Methods

### L-asparaginase Production and Purification

ASNase P40S/S206C (hereinafter referred to as ASNase), was expressed in an *Escherichia coli* BL21 (DE3) strain transformed with plasmid pET 15b encoding the recombinant enzyme [5]. Bacterial stocks were stored in 8% glycerol and maintained at -80 ºC. After thawing at room temperature for 15 min, 15 µL of their suspension were inoculated directly into 20 mL of Luria Bertani (LB) medium (5 g yeast extract L^−1^; 10 g tryptone L^−1^ and 10 g sodium chloride L^−1^) in a 100 mL Erlenmeyer flask. The inoculated broth was incubated at 37 ºC with shaking at 250 rpm for 16 h. Cells were harvested by centrifugation at 3220 × *g* for 20 min at 4 ºC and reinoculated into 1 L of fresh LB medium in an Erlenmeyer flask at optical density at 600 nm (OD_600nm_) equals to 0.1. The broths were incubated at 37 ºC and 250 rpm. Once an OD_600nm_ between 0.6 and 0.8 had been reached, IPTG was added to the medium at a concentration of 0.3 μM to induce ASNase expression. Cells were harvested after 22 h of induction by centrifugation at 3220 × *g* and 4 ºC for 20 min. The cell pellet was then lysed by osmotic shock; briefly, each gram of wet cell were resuspended in 25 mL of hyperosmotic buffer (Tris/HCl 20 mM, pH 8.0, 1 mM EDTA, 20% sucrose), gently stirred at 4 ºC for 5 min, and recovered by centrifugation at 10,000 × *g* for 15 min at 4 ºC. The resulting pellet was resuspended in 25 mL of an hypoosmotic solution (10 mM MgSO_4_, 0.5 mM phenylmethylsulfonyl fluoride) per gram of wet cell, stirred at 4 ºC for 10 min, and the resulting cell debris were collected by centrifugation at 10,000 × *g* for 30 min at 4 ºC. The cell free extract containing soluble proteins was then filtered through a 0.22 µm pore size PES syringe filter.

Purification of ASNase from other soluble proteins was achieved in two chromatographic steps using a General Electric ÄKTA Purifier 10 FPLC System. In the former step, 300 mL of the crude extract were loaded onto a 5 mL DEAE FF anion exchange column previously equilibrated with Tris/HCL 20 mM (pH 8.0) and eluted in 8 mL of Tris/HCL 20 mM (pH 8.0) plus NaCl 130 mM at a flow rate of 2.5 mL min^−1^ at room temperature. Eluted fractions were collected, combined, and concentrated to 0.5 mL using Amicon Ultra 30.000 Da cutoff by centrifugation at 1600 × *g*, 4 ºC. In the latter step, the concentrated semi-crude extract was subjected to a Superdex Increase 200 10/300 GL size exclusion chromatography, and elution was performed at room temperature over 1.5 column volumes using 10 mM potassium phosphate, pH 7.4 as mobile phase, at 0.3 mL min^−1^ flow rate and monitored by UV absorbance in AKTA Purifier 10 FPLC system. Eluted protein purity was assessed by SDS-PAGE 12%, and its concentration determined by BCA assay [15].

### Synthesis of N-terminal Pegylated L-asparaginase (NT-PEG-ASNase)

Optimisation of ASNase N-terminal pegylation was carried out using 1 mg.mL^−1^ of enzyme in 10 mM potassium phosphate buffer, pH 7.4. N-hydroxylsuccinimide (NHS)-polyethylene glycol (mPEG-NHS) was added at 25:1 or 50:1 PEG-protein ratio and kept at room temperature under 400 rpm agitation using magnetic stirrer (Corning PC-420D, Massachusetts, USA) for 20, 30 or 60 min. To quench the reaction and cleave side reaction adducts, 100 mL hydroxylamine chloride (1 M) was added [16]. Two main variables were assessed for maximizing monopegylated ASNase yields in batch reactions, namely (i) the reaction time (20, 30 and 60 min) and (ii) the PEG-protein molar ratio (25:1 and 50:1).

### Synthesis of Cysteine Pegylated L-asparaginase (Cys-PEG-ASNase)

Cysteine pegylation is only possible when the cysteine residue is reduced. This was achieved by incubating 1 mg.mL^−1^ of ASNase in 10 mM potassium phosphate buffer pH 7.4 with 20 mM tri(2-carboxyethyl)phosphine hydro-chloride (TCEP) for 30 min at room temperature in a rotating tube mixer at 8 rpm (AP22 Phoenix Luferco, Phoenix Ind. and Com Equips Scientific LTDA; Araraquara, SP, Brazil). TCEP was removed from the reaction mixture by ultrafiltration using Amicon Ultra centrifugal filters with 30 kDa cut-off; the reaction mixture was diluted five times with 10 mM phosphate buffer and protein was recovered after centrifugation at room temperature for 8 min at 1500 × *g*. The procedure was repeated six times to ensure complete removal of TCEP before pegylation reaction was carried out. For pegylation reaction, the enzyme was kept in 1 mL of 10 mM phosphate sodium buffer after reduction, and 5, 10 or 25 mPEG-Mal:protein ratio was added. The mixture was stirred at 400 rpm using magnetic stirrer (Corning PC-420D, Massachusetts, USA) at room temperature for 30 or 60 min. Reactions were quenched by dilution in 5 mL of 10 mM potassium phosphate buffer pH 7.4 followed by ultrafiltration in Amicon centrifugal filters of 30 kDa cut-off (centrifugation at room temperature for 8 min at 1500 × *g*) to remove unreacted mPEG-MAL and byproducts; this step was carried out 6 times to ensure complete removal of mPEG-MAL. Two variables affecting monopegylation yields were studied, namely (i) the reaction time (30 and 60 min) and (ii) the PEG-protein ratio (5:1, 10:1, and 25:1).

### Purification of Pegylated ASNase

The reaction mixtures were concentrated to 500 μL by ultracentrifugation at 1.500 xg, 4 ºC using Amicon 30 kDa cut-off filters. Protein samples were purified by size exclusion chromatography (SEC) using a General Electric ÄKTA Purifier 10 FPLC system fitted with a Superdex Increase 200 10/300 GL column. Protein was eluted with 10 mM potassium phosphate buffer pH 7.4 as the mobile phase at a flow rate of 0.3 mL min^−1^. Elution was followed by absorption of the flow-through at UV_280nm_, and reaction yields were calculated by peak integration. Fractions of 500 μL were collected and stored at 4 ºC for further analysis.

### Native-Electrophoresis

Electrophoresis (Native-PAGE) was used to assess if the attachment of mPEG on the structure of ASNase was successful. Samples were mixed in a 3:1 ratio with Tris/HCl loading buffer, pH 6.8, 0.2% (w/v) bromophenol blue, 20% (v/v) glycerol, and a final volume of 15 µL was loaded onto the stacking gel. The resolving gel was composed of 522 mM Tris/HCl, pH 8.8, 6% (w/v) acrylamide/bis-acrylamide mix, 0.09% (w/v) PSA and 0.19% (v/v) tetrametiletylenediamine (TEMED). The stacking gel was composed of 116 mM Tris/HCl, pH 6.8, 5.0% (v/v) acrylamide/bis-acrylamide mix, 0.14% (w/v) PSA and 0.29% (v/v) TEMED. Electrophoresis was carried out in a running buffer of 25 mM Tris/HCl, 192 mM glycine, pH 8.3, at 120 V for 100 min. Gels were stained with 0.25% (w/v) Coomassie G-250, 45% (v/v) methanol and 10% (v/v) acetic acid for 40 min at room temperature under orbital shaker Sunflower 3D Mini-Shaker (Biosan, Latvia), followed by destaining by soaking the gel overnight at room temperature in a solution containing 45% (v/v) methanol and 10% (v/v) acetic acid and gently mixed at Sunflower 3D Mini-Shaker (Biosan, Latvia).

### LC–MS/MS analysis of Conjugates

One μg of trypsin (Bovine, Cat. No. 000000011047841001; Sigma) was added to the samples at a ratio of 1:10 enzyme:substrate in phosphate buffer 10 mM, pH 7.4 and incubated at 37 °C overnight in a shaking heat block at 750 rpm. Samples were dried to completion in a Speedvac (Thermo Fisher Scientific) and prepared for peptide clean up using C18 spin columns (#89,852; Thermo Fisher Scientific) by resuspension in 300 μL of 0.1% (v/v) trifluoroacetic acid (TFA). Samples were prepared according to manufacturer’s instructions and eluted in 50% (v/v) acetonitrile and 0.1% (v/v) TFA, dried to completion in a Speedvac and stored at -80 °C.

The extracted peptide samples were resuspended in 20 μL of MS sample buffer (2% (v/v) acetonitrile in 0.05% (v/v) formic acid) (Solution A), 6 μL of which were injected into the LC–MS/MS system, equivalent to 3 μg on the column. Chromatographic separation was performed using a U3000 UHPLC NanoLC system (ThermoFisherScientific, UK). Peptides were resolved by reversed phase chromatography on a 75 μm C18 Pepmap column (50 cm length) using a three-step linear gradient of 80% (v/v) acetonitrile in 0.1% (v/v) formic acid (Solution B). The gradient was delivered to elute the peptides at a flow rate of 250 nL min^−1^ over 60 min, starting at 5% (v/v) solution B (0–5 min) and increasing solvent to 40% (v/v) solution B (5–40 min) prior to a wash step at 99% (v/v) solution B (40–45 min) followed by an equilibration step at 5% B (v/v) (45–60 min).

The eluate was ionised by electrospray ionisation using an Orbitrap Fusion Lumos (ThermoFisherScientific, UK) operating under Xcalibur v4.3. The instrument was first programmed to acquire data using an Orbitrap-Ion Trap method by defining a 3 s cycle time between a full MS scan and MS/MS fragmentation by collision induced dissociation. Orbitrap spectra (FTMS1) were collected at a resolution of 120,000 over a m/z scan range of 375–1800 with an automatic gain control (AGC) setting of 4.0e5 (100%) with a maximum injection time of 35 ms. Monoisotopic precursor ions were filtered using charge state (+ 2 to + 7) with an intensity threshold set between 5.0 × 10^3^ to 1.0 × 10^20^ and a dynamic exclusion window of 35 s ± 10 ppm. MS2 precursor ions were isolated in the quadrupole set to a mass width filter of 1.6 m/z. Ion trap fragmentation spectra (ITMS2) were collected with an AGC target setting of 1.0e4 (100%), a maximum injection time of 35 ms, and CID collision energy set at 35%.

Raw mass spectrometry data were processed into peak list files using Proteome Discoverer (ThermoScientific; v2.5). These raw data files were searched using the Sequest [17] search algorithm against the Uniprot All Taxonomy database (569,516 entries). Database searching was performed at a stringency of 1% FDR including a decoy search. Posttranslational modifications for carbamidomethylation (Cys), oxidation (Met), dioxidation (Cys), trioxidation (Cys) and acetylation (Lys and N-terminal) were included in the database search as variable.

### Determination of L-asparaginase Activity

Enzymatic activity was determined using the Nessler assay modified by Simas et al. [18]. Briefly, 10 μL of enzyme samples were incubated for 30 min in 96-well plates with 100 μL of 50 mM Tris/HCl pH 8.6, 80 μL of ultrapure water, and 20 μL of 100 mM L-asparagine. After incubation for 30 min at 37 ºC in an incubator (Quimis Q316M4, Diadema, Brazil), the reaction was quenched with 10 μL of 1.5 M trichloroacetic acid. Next, 35 μL of the reactional mixture was diluted with 280 μL ultrapure water and 35 μL of Nessler reagent (Merck, Germany) and kept at room temperature for 10 min under no agitation. Absorbance was then measured in a SpectraMax Plus 384 at 436 nm. Blanks were carried out containing ultrapure water instead of L-asparagine. All measurements were performed in triplicate, and results were expressed as average values ± standard deviation. Specific activity was calculated by dividing the activity value by the protein concentration obtained from the BCA method [15]. Activity results were expressed in U, meaning the quantity of enzyme needed to convert 1 μmol of substrate in a minute at the given reaction conditions.

### Determination of Kinetics Parameters

Kinetics parameters of free and pegylated forms of ASNase were assessed using an assay described by Balcão et al. (2021). Using 96-well UV acrylic microtiter plates (Corning, USA), reaction mixtures of 131 µM NADH, 17.5 µL of 400 mU bovine glutamate dehydrogenase (GDH) (Sigma Aldrich, United States of America), 3.5 µL of 1 mM α-ketoglutarate, and 159 µL of L-asparagine (at concentrations of 4, 2, 1, 0.75, 0.5, 0, 25 and 0.1 mM) were added at a final volume of 300 µL per well buffered with 87.5 µL of 50 mM Tris/HCl, pH 8.0. The microtiter plates were incubated at 37 ºC for 5 min before addition of 50 µL of 50 nM pre-heated (37 ºC, 10 min) ASNase. Reactions were carried out at 37 ºC. Every experiment was repeated four times.

ASNase catalyzes the hydrolysis of asparagine to aspartate and ammonia. This ammonia can be condensed with α-ketoglutarate in an NADH dependent reaction catalysed by GDH, releasing NAD^+^ which was measured at 340 nm at 15 s intervals for 10 min. The absorption values were transformed using Beer-Lambert’s law (NADH molar efficient coefficient = 6.1 µM^−1^ cm^−1^) to provide velocity vs L-asparagine concentrations plots. Michaelis-Mentem kinetics were assessed using Graphpad Prism 5 software to determine values of *V*_*max*_ and *k*_*M*_.

### Thermodynamic Study of Free and Pegylated L-asparaginase

The specific activities of ASNase, NT-PEG-ASNase and Cys-PEG-ASNase at 0.025 mg protein mL^−1^ in 50 mM Tris/HCl buffer, pH 8.6, were determined at 20, 30, 40, 50, 60, 70, 80, and 90 ºC every two minutes as described in Sect. 2.6. Two Arrhenius plots were drawn, one displaying enzyme activity increase versus temperature (*T* < *T*_opt_), and the other decreasing activity versus temperature (*T* > *T*_opt_). The slope of the ascending portion of Arrhenius linear equation (Eq. 1) is equal to –*E*_*a*_/*R*, which was used to calculate the activation energy (*E*_*a*_, kJ mol^−1^) of the reaction, i.e., ASN cleavage:

1 $$\mathrm{lnA}=-\frac{Ea}{RT}+\mathrm{ln}a{\prime}$$

where *A* is the enzyme activity (U), *R* is the universal gas constant (8.314 J.K^−1^.mol^−1^), *T* is the temperature (K) and ln *a*^*’*^ is the intercept of the straight line. From the activation energy, the enthalpy variation (Δ*H*) involved in the formation of the enzyme–substrate complex (E-S) was calculated using Eq. 2:

2 $$\Delta H=Ea-RT$$

The lower the *E*_*a*_ and Δ*H* values, the higher the activity of an ideal enzyme [19]. In the ascending portion of Arrhenius plot, i.e., at *T* > *T*_opt_, equilibrium is shifted towards the unfolded state of the enzyme. Therefore, the energy related to the change in enzyme state is the variation of enthalpy of unfolding equilibrium Δ*H*_*u*_^*o*^, which can be calculated from the slope of the straight line of the descending portion of the Arrhenius plot (Eq. 3):

3 $$\mathrm{ln}A=\frac{{\Delta H}_{u}^{o}-{E}_{a} }{R}\bullet \frac{1}{T}+\mathrm{ln}\frac{a{\prime}}{b}$$

where* b* is another pre-exponential factor.

### Thermodynamic Study of ASNase and PEG-ASNase Thermal Inactivation

The specific activities of ASNase, NT-PEG-ASNase and Cys-PEG-ASNase at 0.025 mg protein mL^−1^ in 50 mM Tris/HCl buffer, pH 8.6, were determined at 75, 76, 77, 78, 79 and 80 ºC every two minutes as described in Sect. 2.6. Values of ASNase activity before heating were taken as activity (*A*_o_) at the beginning (*t*_0_ = 0). Decay of activity can be understood as a first order reaction, therefore, a first order constant of denaturation *k*_*d*_ can be calculated according to Eq. 4:

4 $$\mathrm{ln}\left(\frac{A}{Ao}\right)= -kd t$$

where *t* is the time.

The variations of Gibbs free energy (Δ*G*_*d*_, Eq. 5), enthalpy (Δ*H*_*d*_, Eq. 6) and entropy (Δ*S*_*d*_, Eq. 7) of enzyme denaturation can then be calculated as:

5 $${\Delta G}_{d}= -RT\mathrm{ln}\left(\frac{{k}_{d}h}{{k}_{B}T}\right)$$

6 $${\Delta H}_{d}= {E}_{d}-RT$$

7 $${\Delta S}_{d} =\frac{{\Delta H}_{d} - {\Delta G}_{d}}{T}$$

where *k*_*B*_ and *h* are the Boltzmann constant (1.38 × 10^23^ J.K^−1^) and the Planck constant (6.62 × 10^–34^ J.s), respectively, while *E*_*d*_ is the activation energy of denaturation (kJ mol^−1^).

The values of time of decimal reduction *D* (s), defined as the time taken to reduce the activity by thermo-inactivation to 90%, were calculated according to Eq. 8:

8 $$D =\frac{\mathrm{ln}10}{kd}$$

The resistance to temperature increase, *Z* (ºC), which is defined as the increment in temperature that leads to a 90% reduction of *D*, was calculated by linear regression of log *D* vs *T* (Eq. 9):

9 $$Log D= -\frac{1}{Z} \left(T-Tref\right)+\mathrm{log}Dref$$

where Tref is the lowest set temperature and *Dref* is the corresponding *D*-value.

## Results

### L-asparaginase Pegylation

**Fig. 1:**
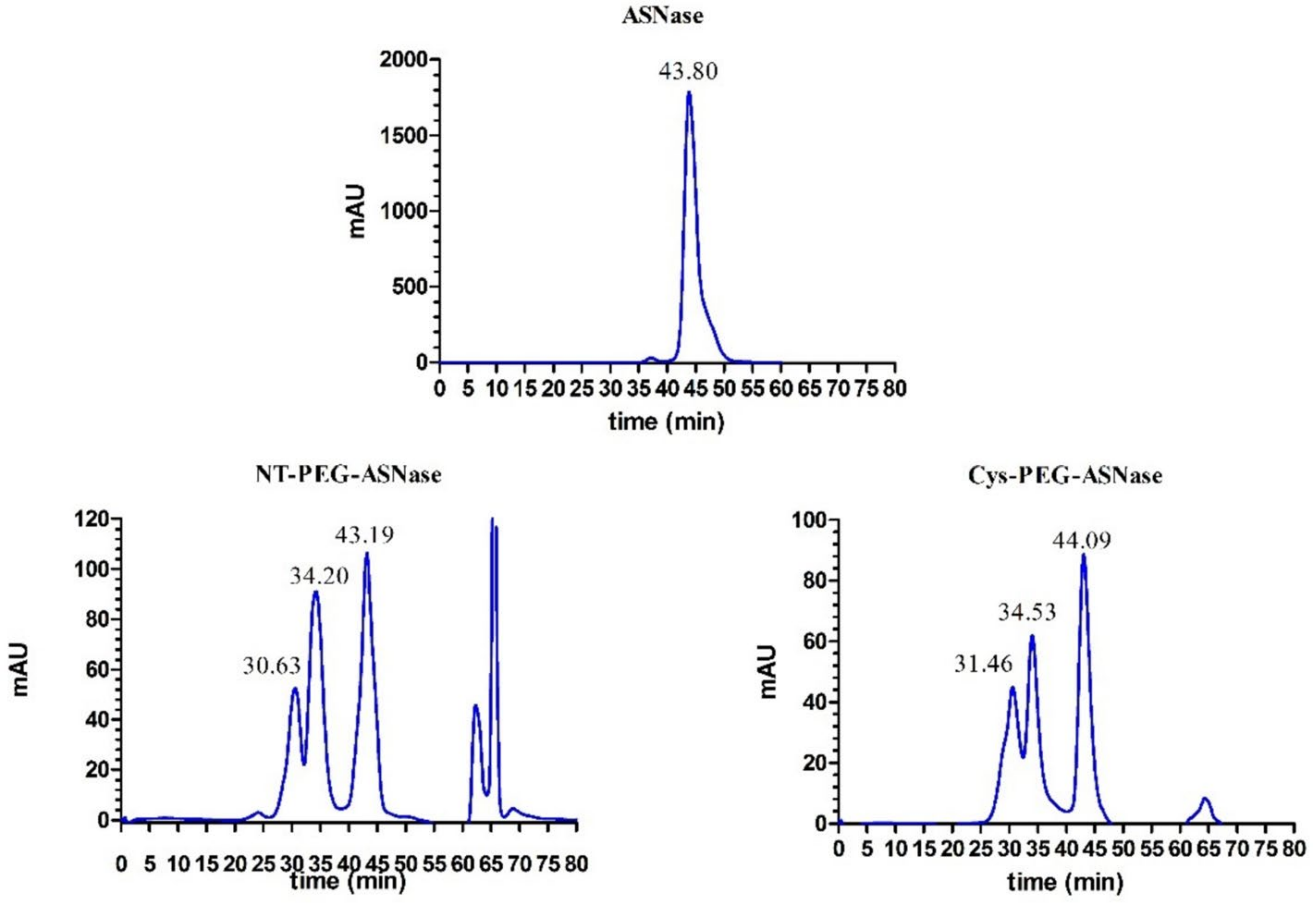
Chromatogram (size exclusion chromatography) of the pegylation reactions. Blue lines represent the absorbance at 280 nm. Numbers above the peaks are retention times (minutes). Peaks above 60 min reffer to unreacted mPEG-NHS or mPEG-MAL and byproducts from the conjugation reaction. The order of elution of mPEG-NHS and mPEG-Mal reactions was polypegylated, monopegylated and non-pegylated species

ASNase was produced, purified, and allowed to react with mPEG-NHS and mPEG-Mal, and the conjugates were then purified. Since SEC is a separation technique based on differences in molecular size (kDa) among proteins, the reduction in the retention volume to values below 43.80 min (corresponding to non-pegylated ASNase) resulting from the contact between ASNase and the reagent PEG (20 kDa) is indicative of the successful conjugation of the protein with mPEG [20]. Three main peaks were observed in the chromatogram of both pegylation reactions (Fig. 1), whereas the obtained retention times were similar (Table 1), with elution order following the expected theoretical molecular weights (Mw), i.e., Mw > 220 kDa for polypegylated, ~ 220 kDa for monopegylated and ~ 140 kDa for non-pegylated enzyme.

**Table 1:** Reaction yields of ASNase N-terminal pegylation with 20 kDa mPEG-NHS and Cys 206 with 20 kDa mPEG-Mal 20 kDa obtained at different PEG-protein molar ratios and reaction times (minutes)

PEG-protein ratio	Reaction time (min)	Reaction Yield (%)		
		Polypegylated	Monopegylated	Non-pegylated
Pegylation with mPEG-NHS 20 kDa				
25:1	30	14	41	45
25:1	60	15	38	46
50:1	20	15	42	45
50:1	30	38	29	32
50:1	60	87	–-	13
Pegylation with mPEG-Mal 20 kDa				
5:1	30	6	24	70
5:1	60	5	25	69
10:1	30	19	38	54
10:1	60	36	39	25
25:1	30	83	–-	17
25:1	60	83	–-	18

Reactions were carried out in 10 mM phosphate buffer, pH 7.4 at room temperature, 400 rpm agitation and quenched by hydroxylamine addition (mPEG-NHS 20 kDa) and ultrafiltration (mPEG-Mal 20 kDa). Reaction yields were calculated by chromatogram peak integration after size exclusion chromatography in a Superdex Increase 200 10/300 GL column

Some factors are widely known to influence N-terminal site-specific pegylation such as the pH, mPEG/protein molar ratio, temperature, and buffer composition [21, 22]. Here, the PEG-protein molar ratio and reaction time were investigated, and the results are summarized in Table 1.

The yield of pegylation reaction with mPEG-NHS was influenced by both reaction time and PEG/protein molar ratio. When ASNase was pegylated with 50 equivalents of mPEG-NHS, the reaction yield was dependent on the reaction time before quenching with hydroxylamine. Within 20 min, 42% of the enzyme was converted into NT-PEG-ASNase, while an increase in reaction time to 30 min led to more conjugation not only in the non-reacted enzyme but also in the monopegylated species, resulting in no less than 87% of polypegylated enzyme after 60 min. Decreasing the PEG/protein ratio to 25, 41% of the enzyme was found monopegylated, but no significant increase in the reaction yield was found by increasing the reaction time from 30 to 60 min. Since the best yield of monopegylated enzyme was around 40% in two conditions, the 25 PEG/protein molar ratio in 30 min was considered a better outcome compared to the 50 one in 20 min, which provided a smaller amount of mPEG-NHS to be employed.

Given that polypegylation was also observed for mPEG-MAL conjugation, by observation of a peak in the chromatogram at 31.46 min (Fig. 1) similar to that at 30.63 min in mPEG-NHS conjugation, PEG-protein ratios and reactions times were screened to increase Cys-PEG-ASNase yield, providing the results summarized in Table 1. At 5:1 PEG/protein ratio, i.e., close to the stoichiometric proportion considering the tetrameric nature of ASNase, an increase in the reaction time from 30 to 60 min had no effect on the conjugation yield (25%), and the same occurred using an excess of mPEG-Mal (25:1). The yield was only time dependent at a middle level of the PEG/protein ratio (10:1), at which a raise in the reaction time from 30 to 60 min increased the polypegylation yield from 19 to 36%, whilst keeping the monopegylation yields at 39%. Therefore, a PEG/protein ratio of 10:1 and 30 min reaction time were selected as the optimal condition for Cys-pegylation.

It is noteworthy that mPEG-MAL was more effective in conjugating ASNase than mPEG-NHS, as at a PEG/protein ratio as low as 10:1 it performed (nearly 40% of Cys-PEG-ASNase) similarly to mPEG-NHS at a much higher PEG/protein ratio (25:1).

**Fig. 2:**
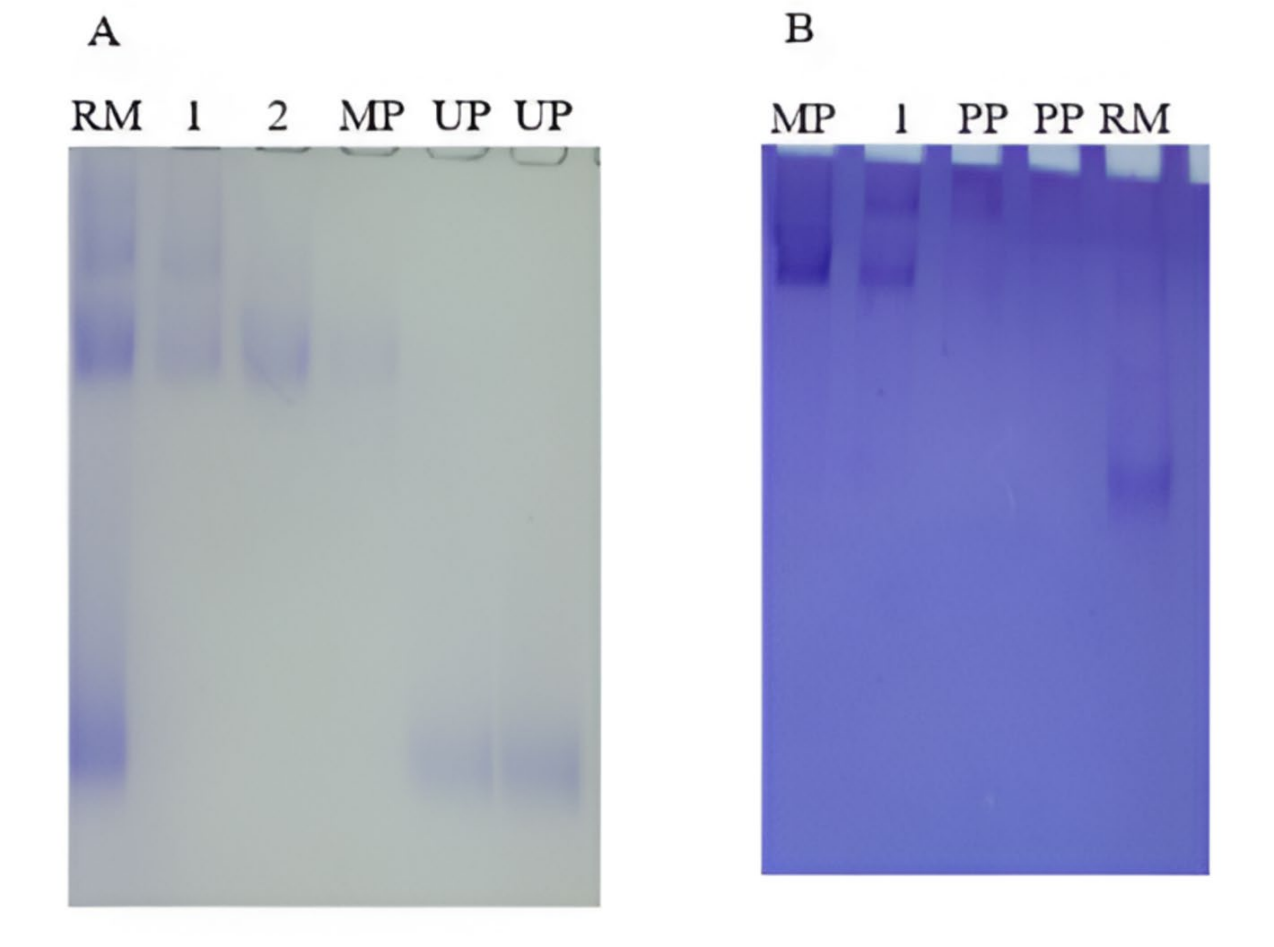
Gel electrophoresis (Native PAGE) of (**A**) N-terminal pegylated ASNase. Lanes: RM: reaction mixture; 1 and 2: purification fractions containing both monopegylated and polypegylated forms; MP: monopegylated form; UP: unreacted protein. (**B**) Cys pegylated ASNase. Lanes: RM: Reaction mixture; PP: polypegylated; 1: purification fractions containing both monopegylated and polypegylated; MP: monopegylated

An electrophoresis analysis (native-PAGE) of the different fractions from pegylated ASNase purification was performed to confirm the conjugation (Fig. 2); however, for Cys-pegylation significant smearing was observed (Fig. 2B). Electrophoretic analyses of pegylated proteins also pose several challenges such as poor penetration of protein conjugates across polyacrylamide gel due to hindrance effects and increase in solvation [23].

**Fig. 3:**
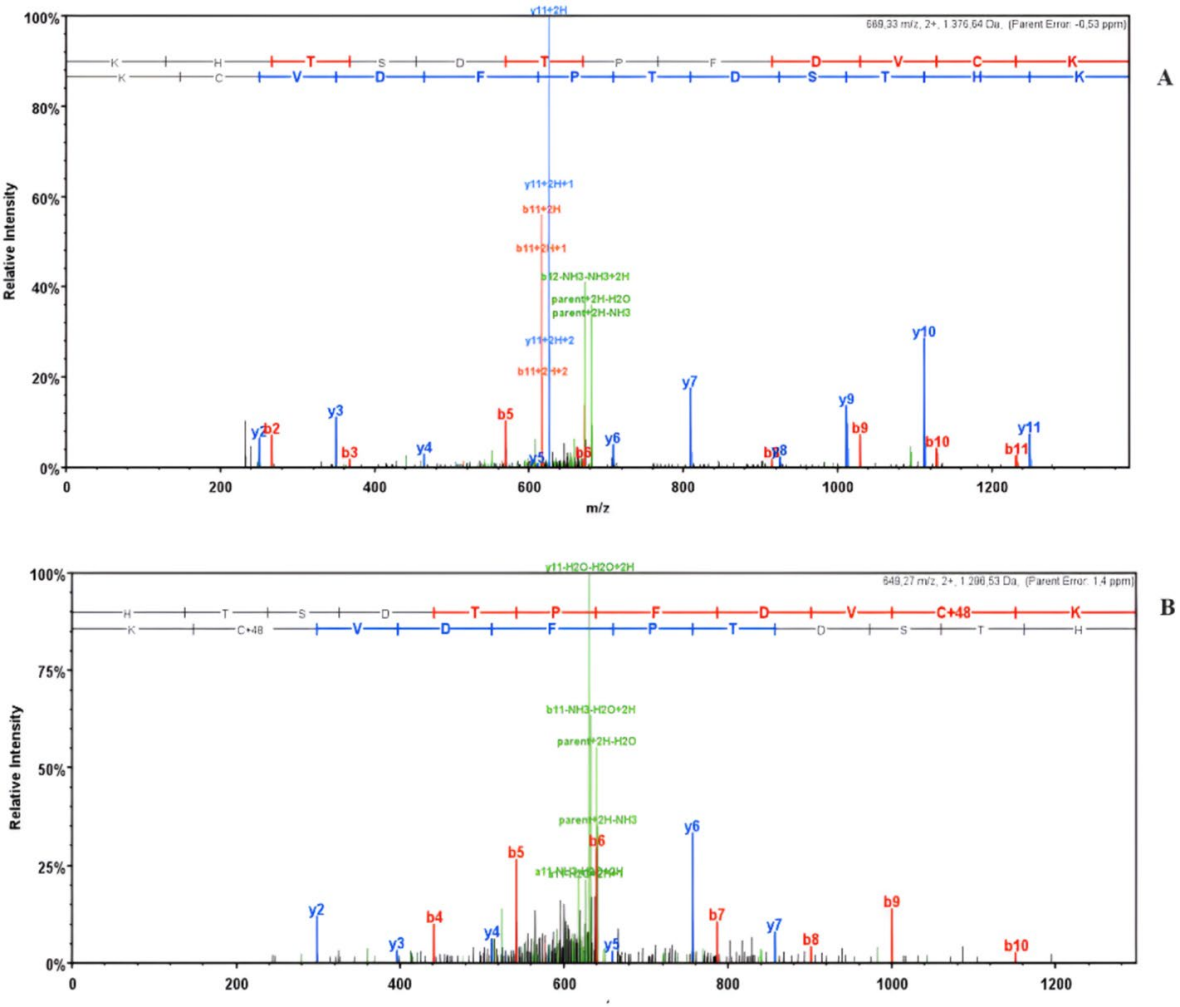
(**A**) Fragmentation spectra evidence for a database showing no cysteine oxidation on the peptide ^196^KHTSDTPFDVCK^207^ with m/z 689.33^2+^. Supporting Cys206 as pegylation site. (**B**) Fragmentation spectra evidence for a database assigned trioxidation modification on the peptide ^197^HTSDTPFDVC_triox_K^207^ with m/z 649.27^2+^. Correct assignment to the b6-ion determines location of trioxidation to the Cys206 residue

Pegylation with mPEG-NHS of N-terminal Leu present in *E.coli* L-asparaginase II variants has been previously established and characterized in a previous work by our group [13], with the position of PEG being identified by MALDI-TOF. However, site specific pegylation of cysteine on the mutant ASNase P40S/S206C had not previously been determined. Since Cys is prone to reduction, DTT could cause removal of the conjugation and leave all the Cys in the reduced state. To avoid this, DTT was not used, 1 mg trypsin mL^−1^ was applied, and digestion was kept overnight to obtain the digested peptides. The tri-oxidated forms at Cys 77 and Cys 105 without reduced forms were identified (Supplementary files). On the contrary, Cys 206 was found to be reduced (Fig. 3A), suggesting this site is favorable for mPEG-Mal conjugation. Although Cys 206 was identified as the site of pegylation, tri-oxidated forms of this residue were also found (Fig. 3B), suggesting incomplete conjugation and that not all four subunits of ASNase were pegylated or the occurrence of retro-Michael reactions [24] displacing the conjugation.

### Effect of Pegylation Site On the Activity of ASNase

**Fig. 4:**
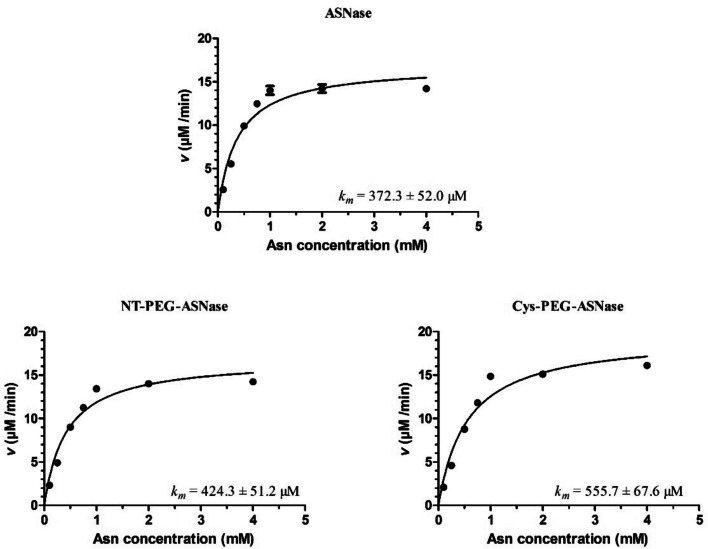
Kinetics of Asn cleavage activity of ASNase, NT-PEG-ASNase and Cys-PEG-ASNase based on GDH coupled assays. Velocities (µM min.^−1^) were plotted as a function of initial Asn concentration. Data was fitted to the Michaelis–Menten model to estimate *k*_*m*_ values (Graphpad Prism 5.0)

ANOVA analysis was used to highlight possible differences among the specific activities (Table 2) of ASNase, NT-PEG-ASNase and Cys-PEG-ASNase (*p* < 0.05). According to Tukey’s test, ASNase had similar activity to NT-PEG-ASNase and Cys-PEG-ASNase (*p* = 0.1173 and* p* = 0.0975, respectively), but the activity of Cys-PEG-ASNase (43.0 ± 1.6 U mg^−1^) was statistically significantly higher (*p* < 0.05) than that of NT-PEG-ASNase (36.2 ± 2.4 U mg^−1^). The higher activity of Cys-PEG-ASNase was also confirmed by GDH coupled assay (Fig. 3) used to assess enzyme kinetics; Cys-PEG-ASNase had higher *V*_*max*_ (19.52 ± 0.80 µM min^−1^) than both ASNase (16.91 ± 0.68 µM min^−1^) and NT-PEG-ASNase (16.88 ± 0.59 µM min^−1^), but its affinity for substrate (*k*_*M*_ = 555.7 ± 67.6 µM) was the lowest (Table 2 and Fig. 4).

**Table 2:** Specific activity and thermodynamics parameters of ASNase, NT-PEG-ASNase and Cys-PEG-ASNase

	ASNase	NT-PEG-ANSase	Cys-PEG-ASNase	*p*-value
*k*_*M*_ (µM)	372.3 ± 52.0	424.3 ± 51.2	555.7 ± 67.6	< 0.05
*V*_*max*_ (µM min^−1^)	16.91 ± 0.68	16.88 ± 0.59	19.52 ± 0.80	< 0.05
Specific activity (U mg^−1^)	39.5 ± 0.5	36.2 ± 2.4	43.0 ± 1.6	< 0.05
*E*_*a*_ (kJ mol^−1^)	19.39 ± 0.18	19.56 ± 0.38	18.69 ± 0.17	< 0.05
Δ*H*_*70 ºC*_ (kJ mol^−1^)	19.20 ± 0.34	18.91 ± 0.75	16.20 ± 0.53	< 0.05

Specific activities were calculated by the modified Nesslerization method and calculated as U/mg, where 1U expresses the amount of enzyme needed to convert 1 μmol Asn to 1 μmol NH_3_ per minute at 37 ºC. Kinetic parameters *V*_*max*_ and *k*_*m*_ were calculated by GDH-NADH coupled assay applying Michaelis–Menten model. Thermodynamic parameters activation energy (Ea) and enthalpy variation at 70 ºC (Δ*H*_*70 ºC*_) were based on the Arrhenius linear equation and enthalpy of E-S complex formation equation, respectively; in both cases the lower the value, the higher is the enzyme activity. Values are expressed as mean values ± standard deviation. *p*-values were calculated from ANOVA test

### Thermodynamic Study of Enzyme Activity and Stability

**Fig. 5:**
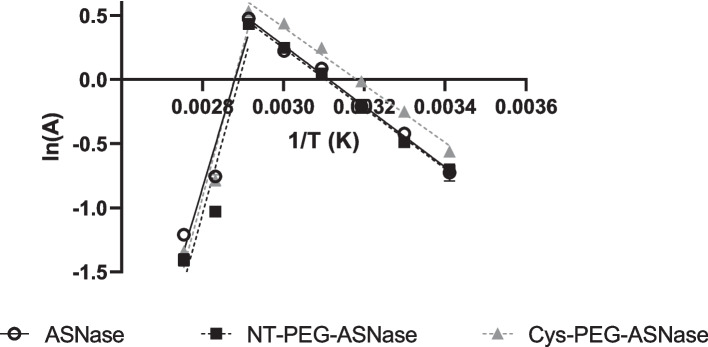
Arrhenius plot for the activity of ASNase (ascending straight line: R^2^ = 0.9931, descending straight line: R^2^ = 0.9575), NT-PEG-ASNase (ascending straight line: R^2^ = 0.9974, descending straight line: R^2^ = 0.9032) and mono-PEG-Cys-PEG-ASNase (ascending straight line: R^2^ = 0.9856, descending straight line: R^2^ = 0.9529). Activities were determined by modified Nesslerization method at different temperatures (20, 30, 40, 50, 60, 70, 80, and 90 ºC) and plotted against the inverse of its corresponding temperature (1/T). The error bars correspond to standard deviation

Based on the enzyme activity measurements at different temperatures, Arrhenius semi-log plots of ln*A* vs. 1/*T* were built, and two segments could be observed (Fig. 5). In the first one, from 20 to 70 ºC, the increase in temperature led to an increase in ASNase activity for all forms of the enzyme, either pegylated or not. In the second segment, from 70 to 90 °C, denaturation took place, and the increase in temperature was responsible for an activity decrease. Based on the Arrhenius plots, the activation energy (*E*_*a*_, kJ mol^−1^) and enthalpy of E-S complex formation at *T*_opt_ (Δ*H*_*70 °C*_, kJ mol^−1^) were calculated (Table 2).

The values of *E*_*a*_ and Δ*H*_*70 ºC*_ showed the same trend observed for the specific activity with no statistically significant difference between ASNase and Cys-PEG-ASNase (*p* = 0.1027), but with statistically significant difference (*p* < 0.05) between the two pegylated forms. Particularly, *Ea* of NT-PEG-ASNase (19.56 ± 0.38 kJ mol^−1^) was higher than that of Cys-PEG-ASNase (18.69 ± 0.17 kJ mol^−1^), consistently with the previously discussed reduction of the N-terminal pegylated enzyme activity. In fact, since both thermodynamic parameters are correlated with the formation of E-S complex, the lower their values, the more effective the biocatalyst [25].

At temperatures above *T*_opt_ (70 ºC), the starting activity of an enzyme decreases due to unfolding of its tertiary structure. The descending tract of the Arrhenius plot (Fig. 5) allows estimating the standard enthalpy change of unfolding equilibrium (Δ*H*_u_^o^), which is an indirect measure of the unfolded state stability, meaning that proteins with higher Δ*H*_u_^o^ values are less prone to undergo unfolding [26]. Pegylation at N-terminal and Cys increased (*p* < 0.05) Δ*H*_u_^o^ by about 5.5% (93.96 ± 0.33 kJ mol^−1^) and 8.2% (96.20 ± 1.09 kJ mol^−1^) (Table 3) compared to the non-pegylated ASNase (87.80 ± 1.13 kJ mol^−1^), thereby highlighting an increased enzyme thermo-stability.

**Table 3:** Thermodynamic parameters of ASNase, NT-PEG-ASNase and Cys-PEG-ASNase thermo-inactivation

	***T* (ºC)**	***k***_***d***_** (min**^**−1**^**)**	Δ***G***_***d***_** (J mol**^**−1**^**)**	Δ***H***_***d***_** (J mol**^**−1**^**)**	Δ***S***_***d***_** (J mol**^**−1**^** k**^**−1**^**)**	***D* (s)**	***Z* (ºC)**	**Δ*H***_***u***_^***o***^** (kJ mol**^**−1**^**)**
ASNase	75	0.5056	88,207	79,337	-59.4	273	29	87.80 ± 1.13
	76	0.5241	88,362	79,329	-59.8	264		
	77	0.6238	88,112	79,320	-59.0	221		
	78	0.6630	88,192	79,312	-59.2	208		
	79	0.6969	88,303	79,304	-59.4	198		
	80	0.7329	88,412	79,296	-59.7	189		
NT-PEG-ASNase	75	0.3791	89,045	67,696	-95.0	364	35	93.96 ± 0.33
	76	0.4124	89,062	67,688	-94.9	335		
	77	0.4293	89,206	67,679	-95.2	322		
	78	0.4363	89,421	67,671	-95.6	317		
	79	0.4970	89,299	67,663	-95.1	278		
	80	0.5394	89,317	67,654	-95.0	256		
Cys-PEG-ASNase	75	0.3784	89,051	32,976	-194.2	365	66	96.20 ± 1.09
	76	0.3757	89,334	32,968	-194.6	368		
	77	0.3931	89,464	32,959	-194.5	351		
	78	0.4219	89,519	32,951	-194.2	327		
	79	0.4177	89,811	32,943	-194.6	331		
	80	0.4467	89,874	32,934	-194.4	309		

*k*_*d*_ values are assigned to each enzyme at a given temperature as the first-order constant of denaturation. Δ*G*_*d,*_ Δ*H*_*d,*_ and Δ*S*_*d*_ were calculated by the second-law of thermodynamics, where positive values of the variation of free Gibbs energy (Δ*G*_*d*_) express denaturation as a non-spontaneous phenomenon, the higher its value, the higher is the thermostability at a given temperature. Δ*H*_*d*_ is the enthalpy variation that takes place upon denaturation, positive values states the denaturation process as endothermic. Δ*S*_*d*_ is defined as the changes in system’s order and positive values are interpreted as loss of system’s disorder. *D*-value can be defined as the amount of time needed to reduce activity to 10%. *Z* (ºC) and Δ*H*_u_^o^ (kJ/mol) are absolute thermodynamic parameters; the first can be defined as the temperature increase to reduce the *D*-value in 90%, while Δ*H*_*u*_^*o*^ is described as the standard enthalpy variations at equilibrium unfolding

**Fig. 6:**
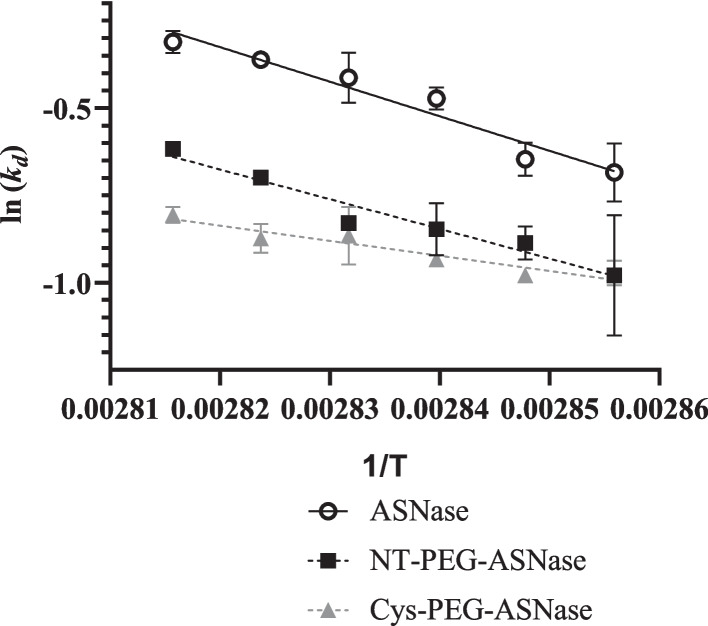
Semi-log plots of the denaturation constant (*k*_*d*_) as a function of the inverse of reciprocal temperature (1/*T*) (K^−1^) for ASNase (R^2^ = 0.9479), NT-PEG-ASNase (R^2^ = 0.9515) and Cys-PEG-ASNase (R^2^ = 0.9100). The slopes of straight lines were used to estimate the enthalpy variation (Δ*H*_d_) of irreversible inactivation. The error bars correspond to standard deviations

While Δ*H*_*u*_^*o*^ describes the process of reversible unfolding, other thermodynamic parameters are used to assess the process of irreversible denaturation throughout the time [27]. In particular, the Arrhenius-type plots of ln*k*_d_ vs 1/*T* depicted in Fig. 6 allowed estimating the variations of Gibbs free energy (Δ*G*_*d*_), enthalpy (Δ*H*_*d*_) and entropy (Δ*S*_*d*_) of the irreversible thermoinactivation for the three investigated forms of ASNase, whose values are listed in Table 3.

The Δ*G*_*d*_ values for the investigated enzyme forms are not the same at a given temperature, due to differences in thermoresistance [28]. The observed increase in Δ*G*_*d*_ at 75 ºC (ΔΔ*G*_*d*_ = Δ*G*_*dPEG-ASNase*_—Δ*G*_*dASNase*_ 840 J mol^−1^) upon pegylation, regardless of the pegylation site, confirms the positive effect of pegylation on protein thermostability. However, at 80 ºC Cys conjugation provided greater thermoresistance (ΔΔ*G*_*d*_ = 1461.4 J mol^−1^) than N-terminal pegylation (ΔΔ*G*_*d*_ = 905.2 J mol^−1^) compared to the free ASNase.

**Fig. 7:**
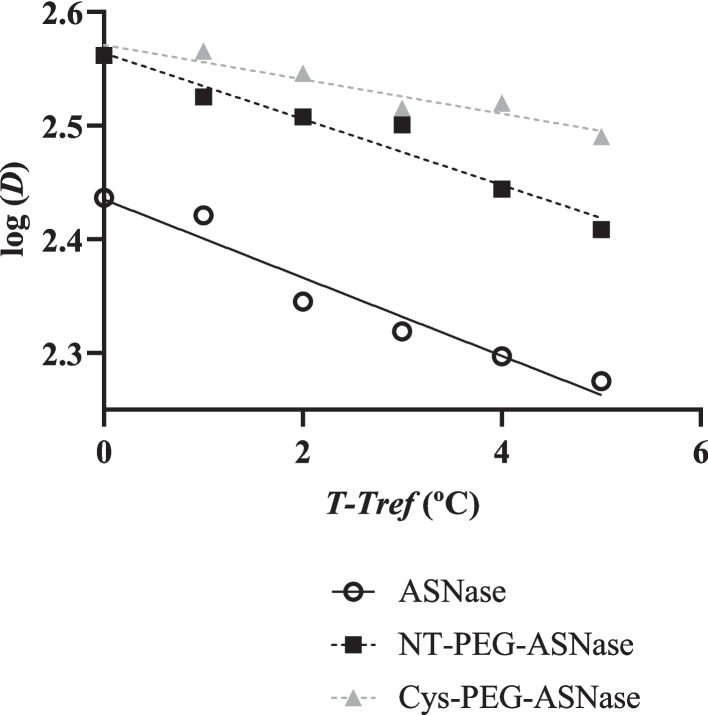
Time of thermal inactivation curves: log (*D*) vs *T*-*T*_*ref*_ (ºC) for ASNase (R^2^ = 0.9464), NT-PEG-ASNase (R^2^ = 0.9491) and Cys-PEG-ASNase (R.^2^ = 0.9071). *T*_*ref*_ is 75 ºC and *D* is the decimal reduction time (s), i.e. the time needed for the activity of the enzyme to be reduced in 90% at a given temperature T (ºC)

The *D*-value is defined as the time taken for an enzyme activity to decay by 90% (10% remaining activity) [29]; therefore, it can also be referred to as decimal reaction time. As can be seen in Table 3, consistently with the increased thermo-stability induced by pegylation, the *D*-values of NT-PEG-ASNase and Cys-PEG-ASNase at 75 ºC were not only almost coincident (364 and 365 s, respectively), but also much higher than that of the free enzyme (273 s). However, the reduction of this parameter with increasing temperature up to 80 °C occurred with higher rate for NT-PEG-ASNase than for Cys-PEG-ASNase (Fig. 7), taking 256 and 309 s, respectively, which confirms the better thermoresistance of the latter form.

From the slopes of the straight lines in Fig. 7 we calculated the *Z*-value, which is conceptually defined as the increase in temperature necessary for a tenfold reduction of the *D*-value and, therefore, can be taken as another indirect measure of the protein thermo-resistance [30]. As expected by the above thermoresistance profile, Cys-PEG-ASNase exhibited a *Z*-value (66 °C) that was higher than that of NT-PEG-ASNase (35 °C) and more than twice that of ASNase (29 °C).

### Long-term Stability of L-asparaginase and Pegylated L-asparaginase

**Fig. 8:**
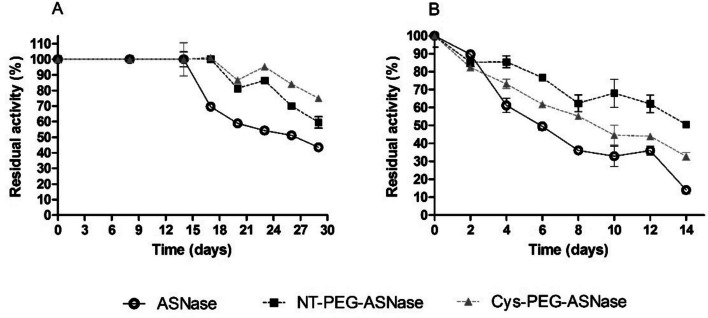
Residual activity of ASNase, NT-PEG-ASNase and Cys-PEG-ASNase along the time at (A) 4 ºC and (B) 37 ºC. Storage in Tris/HCl 50 mM, pH 8.6. Protein concentration was 0.017 mg protein mL^−1^. Activities were calculated by modified Nesslerization method. The error bars correspond to standard deviations

Long-term stability was evaluated at two temperatures, namely 4 ºC and 37 ºC (Fig. 8). No loss of activity was observed up to 14 days at 4 ºC for all the enzyme forms. After 29 days, ASNase was found to keep only 43% of its starting activity, while NT-PEG-ASNase and Cys-PEG-ASNase kept as much as 60 and 75%, respectively, providing a further proof that pegylation, especially Cys conjugation, increased the stability of the enzyme. The same trend was observed at 37 ºC, with ASNase retaining only 14% of its initial activity after 14 days, versus 51 and 33% for NT-PEG-ASNase and Cys-PEG-ASNase, respectively.

## Discussion

Modification of amines by mPEG-NHS can only take place when the amine presents a free pair of electrons; in other words, deprotonated primary and secondary amines are able to undergo conjugation [31]. An in silico analysis using the program H + + [32] and Poisson-Boltzmann server [33] predicted high p*K*_a_ values (> 10.0) for Lys residues in the primary amino acid structure, while the N-terminal residue (Leu1) was predicted to have a p*K*_a_ value between 7.3 and 7.8. Lys301 differed from the other Lys residues in terms of acidity, with a p*K*_a_ of 8.3–8.5 (Supplementary files. Table S1). Reactions were carried out at pH close to the p*K*_a_ of Leu1; therefore, the conjugation occurred primarily on this residue. The p*K*_a_ of Lys301 was close to this, which suggested that side pegylation reactions might also take place on this site leading to poly-PEG-ASNase. At 50:1 PEG-protein molar ratio, the increase in reaction time from 20 to 60 min led to polypegylation of 87% of the enzyme (Table 1). However, the same was not observed when the molar ratio was reduced to 25:1, no significant changes in the pegylation degree being observed between 30 and 60-min reaction times (Table 1). In both cases the yield of monopegylated enzyme was similar (41 and 38%, respectively), and the same was found for polypegylation (14 and 15%). This most likely occurred because the reactive portion of mPEG-NHS is highly susceptible to hydrolysis and, at lower concentrations, degradation was significant even after 30 min, thereby preventing more polypegylation (Table 1).

Cys are among the most targeted residues for site-specific conjugation owing to the low frequency of their exposition in proteins [34]. In addition to being only able to conjugate to maleimide chemicals in a reduced form, they undergo a Michael addition reaction with initial formation of thiolate anion, which results in a nucleophile attack on the π-bond of mPEG-maleimide, leading to a stable thioeter bond [35]. In the case of the ASNase here studied, a free Cys was engineered at the position 206 [5], which is highly exposed to the solvent. Pegylation of ASNase at Cys 206 was analyzed by SEC, and similarly to what was observed in the previous N-terminal pegylation (Fig. 1), the presence of a peak corresponding to polypegylated ASNase (retention time of 31.46 min) in addition to that of the monopegylated form (retention time of 34.53 min) suggests that pegylation might have occurred not only in the free Cys206 but also in the less exposed Cys77 and Cys105 residues, since TCEP is able to reduce Cys and break disulfide bonds. Our proteomics analysis revealed no presence of a Cys77 and Cys105 in its reduced form, meaning it is unlikely that pegylation occurred at the non-mutant Cys residues. We also highlight that mPEG-Mal was more effective in conjugating ASNase than mPEG-NHS at lower PEG-protein ratios due to its higher stability in aqueous systems. In fact, mPEG-NHS hydrolysis occurs rapidly in aqueous systems with half-life of the ester group of about 120 min at room temperature and pH 7.4, while in similar environment mPEG-Mal half-life upon hydrolysis reached 929 min [36, 37].

Even though SEC confirmed that ASNase pegylation increases the Mw and ultimately reduces the retention time from around 43 to 34 min, it is important to keep in mind the limitations of this technique. The decrease in retention time upon pegylation does not typically represent the actual increase in molecular weight [38]; the increase in solvation creates a hydration region that leads to change in the elution behavior across the chromatographic column [39].

The *E*_*a*_ of the reaction catalysed by the wild-type *E. coli* ASNase determined in a previous study was about 30% lower (13.08 kJ mol^−1^) [40] than that found in the present study for non-pegylated mutant ASNase (19.39 ± 0.18 kJ mol^−1^), thereby indicating that the native enzyme is more active than the mutant ASNase, like described by Rodrigues et al. [5], and confirming the Arrhenius model correlation with activity. A study on N-terminal pegylation of wild-type ASNase with a 20 kDa reactive mPEG-propionaldehyde resulted in 57% decrease in the specific activity [41], the same trend was observed for pegylation with mPEG-NHS 10 kDa [13]. However, no such reduction was found in this work when the enzyme was pegylated in its N-terminal residue. The mutant P40S/S206C had its activity previously compared with the wild-type ASNase and a reduction in activity was observed; the mutation P40S promoted an enlargement of a loop in the region of the residue Ser40, which appeared amidst a flexible loop [5]. This enlargement contributed to increase the flexibility of the loop 8–31, which contains the catalytic residues T12 and Y25, leading to a 40% reduction in the activity [42, 43]. Considering that the N-terminal is close to the loop 8–31, conjugation at this site, can be detrimental to the activity due to steric hindrance caused by the PEG chain, as stated by [44] and observed for the mutant enzyme. Given the higher flexibility of the N-terminus close loop (residues 8–31) in the mutant enzyme, the open conformation described by [42] is favoured, probably positioning the PEG chain attached to the N-terminal in a position that causes no hindrance to catalytic residues, conserving 91% of the activity of the non-pegylated mutant enzyme. The decrease in activity is also not correlated to changes in the secondary structure since no influence on enzyme conformation was revealed by Circular Dichroism after pegylation in both sites (Supplementary files. Figure S1).

Other residues described as important for ASNase activity are T89, D90, K162 and E283 [42]. In the case of pegylation with mPEG-Mal, Cys77 and Cys 105 forms the only disulfide bond in the enzyme, which has been previously exploited for pegylation with mPEG-monosulfone, keeping the proximity of both cysteines and conserving the activity [45]. In the case of pegylation with mPEG-Mal, each PEG moiety binds to only one free-thiol cysteine. Therefore, the opening of a disulfide bond between the aforementioned residues following pegylation could likely lead to total loss of ASNase structure. Monopegylation at Cys 206 led to a product with conserved and marginally increased activity, which is also an indicative that Cys 206 is the site of modification, further confirmed by proteomics. A reduction in the enzyme activity is expected when mPEG is attached next to catalytic residues or whenever steric hindrance by the polymer takes place, decreasing the *k*_*M*_ of the enzyme [44]. It was recently reported that low *k*_*M*_ is not essential for antiproliferative activity against ALL cell lines [46], although differences may occur in vivo when Asn bioavailability becomes residual. The *k*_*M*_ increase from 372.3 ± 52.0 µM to 555.7 ± 67.62 µM observed here suggest that even though Cys206 is far from the catalytic residues, the presence of a large mPEG attached to it led to steric hindrance that hindered the access of the substrate to the catalytic site. Nonetheless, no reduction in the specific activity was observed, but rather a slight increase. It is possible that mPEG solvation by water may have stabilized the tetrameric structure of ASNase and made the catalytic residues closer and well positioned for Asn cleavage, leading to an increase in Cys-PEG-ASNase activity.

The variation of Gibbs free energy (Δ*G*_*d*_) is the best tool to assess the thermostability of enzymes, since it measures the combination of entropic and enthalpic contributions to the inactivation of the enzyme. The positive values observed in this work are indicative of a non-spontaneous inactivation, meaning that the enzyme is generally stable at lower temperatures [47]. Thermo-inactivation enthalpy (Δ*H*_*d*_) and entropy (Δ*S*_*d*_) measurements help us to go deeper into the thermo-inactivation phenomenon. Mechanistically, Δ*S*_*d*_ and Δ*H*_*d*_ should be interpreted in combination, the latter meaning actual denaturation process with break of intramolecular bonds, including hydrogen bonds, van der Waals forces, and ionic interactions [26]. The lower Δ*H*_*d*_ calculated for both pegylated forms of ASNase suggests lower thermostability compared to non-pegylated enzyme if we overlook the entropic contribution of thermoinactivation. In turn, negative values of Δ*S*_*d*_ like those listed in Table 3 are indicative of loss of disorder of the system, which suggests aggregation takes place in the solution [26]. It is noteworthy that the less entropic landscape occurred upon denaturation of free ASNase, with 1.6-fold and 3.3-fold more energy per temperature degree being involved in the aggregation of NT-PEG-ASNase and Cys-PEG-ASNase, respectively. The combination of the thermodynamic parameters leads us to interpret that fewer intramolecular bonds break upon heating in Cys-PEG-ASNase in comparison to NT-PEG-ASNase, in exchange for a higher degree of aggregation in the Cys pegylated counterpart. The surface of *E. coli* ASNase is composed of many hydrophobic residues [48], which explains the aggregation behavior here observed for all enzymes.

According to the model established by Meng et al. [49], the stabilization provided by pegylation is dependent on the volume of PEG chains and the exclusion volume (*V*_e_), defined as the volume of the exclusion zone around it formed by the solvating water. Not only the presence of mPEG but also the pegylation site were found to be relevant for ASNase stabilization; so, Cys-PEG-ASNase probably exhibited larger *V*_e_ than NT-PEG-ASNase. Although both conjugates possess the same number of 20 kDa PEG moieties attached, PEG orientation and location at the protein surface may have impacted hydrogen bonds between PEG and protein side chains; in this scenario, less water molecules may have interacted with the PEG corona in the NT-PEG-ASNase, leading to a *V*_e_ reduction [50, 51]. In addition, the PEG corona undergoes dehydration upon increasing the temperature [52], thus resulting in aggregation of pegylated proteins through the hydrophobic effect [53]_._ This occurrence would explain not only the more pronounced decrease in Δ*S*_*d*_ of pegylated forms of ASNase compared to the non-pegylated enzyme, but also why the difference in stability between the two pegylated forms became more pronounced upon increasing the temperature (Fig. 6). Among the residues exposed to the solvent and closer to the pegylation sites (< 10 Å), three of them (Asn 3, Gln 41, Asn 47) are close to the N-terminal and capable to donate hydrogen bonds, therefore could interact with the PEG. On the other hand, five residues close to the Cys 206 (Thr 68, Asn 74, Thr 75, Asn 209, Gln 312) are hydrogen-bond donors. Since more residues around Cys 206 are capable to interact with PEG in comparison to the N-terminal region, this can explain the higher stabilization effect of conjugation at the mutant residue. This observation may conflict with the Meng model, given that more interaction of the PEG chain with the neighbouring residues would lead to less sites to interact with water, reducing the *Ve*. This means that either the Meng model does not fully explain the stabilization effect of pegylation or that PEG hydrogen-bond with the neighbouring amino acids can also lead to changes in the *Ve* by a more complex mechanism. Researchers have been exploring the role of residues in the thermostability of ASNase. [54] showed that mutating the positively charged residues Lys 207 and Lys 139, close to the Cys 206 and Leu 1 respectively, for a neutral amino acid increases ASNase thermostability given that non-desired ionic interactions with those residues were removed. In our work part of the stabilization effect can be explained by the modification of residues close to these positively charged residues, due to steric effect, where the steric effect against Lys 207 by conjugating Cys 206 was higher than pegylating Leu 1 close to the Lys 139. We could confirm this hypothesis by measuring the distance between the residues of Cys 206 and Lys 207 that was found to be 7.4 Å, while the distance between the Leu 1 and Lys 139 residues was found to be 11.1 Å.

The thermodynamic modeling of the enzyme stability explains the long-term stability effects observed when stored at 4 ºC; Cys-PEG-ASNase was found to be stable for to 21 days, and retained 75% of the activity after 30 days, while NT-PEG-ASNase retained 60%. The lower stability of Cys-PEG-ASNase after exposition for 14 days at 37 ºC (33% of activity) compared to NT-PEG-ASNase (51% of activity) may be explained by depegylation via retro-Michael reaction to some extent, where a hydroxyl group would attack the hydrogen in the alpha carbon by a carbonile, the electronic rearrangement would then finally break the bond between the sulfur and the carbon, releasing the PEG from the protein [55], leading to partial loss of the protective effect of PEG.

In the case of pegylated ASNase, the ideal scenario would be a longer-circulating conjugate, given that its target Asn is present in the bloodstream. ASNase would benefit greatly from a higher stable conjugation using alternative linkers, that does not undergo retro-Michael reaction, preventing depegylation. One example would be pegylating the ASNase with mPEG-monosulfone, with a post-treatment with a borohydride in order to protonate the carbonile and prevent the retro-michael reaction, [56]. To prevent retro-Michael reaction in maleimide conjugates, [57] proposed that the opening of the maleimide ring by hydrolysis would lead to a more stable conjugate. For this reason, self-hydrolysing maleimide reagents have been explored in recent years [58]. In both cases, pegylation of ASNase would increase the stability of the conjugate in solution.

## Conclusions

In this paper we described the site-specific pegylation of a novel mutant ASNase in two possible residues, namely N-terminal and Cys206, the latter artificially introduced in the protein structure. Pegylation at Cys residue was more efficient than pegylation at the N-terminal since lower concentrations of reactive mPEG resulted in similar yields. The study of the influence of conjugation site on ASNase activity revealed higher activity of Cys-PEG-ASNase compared to NT-PEG-ASNase, which was confirmed by the thermodynamic study of enzyme activity. Pegylation increased the enzyme thermostability and, in particular, Cys conjugation had a better effect than N-terminal conjugation. The long-term stability of ASNase was also increased, with Cys-PEG-ASNase being more stable than NT-PEG-ASNase at 4 ºC, whilst the opposite trend was observed at 37 ºC.

## Supplementary Information

Below is the link to the electronic supplementary material.

Supplementary file1 (DOCX 179 KB)

## Data Availability

The datasets generated during and/or analyzed during the current study are available from the corresponding author on reasonable request.
