# Supplementary material for: Cysteine Pegylation of a Mutant L-asparaginase Affords Enhanced Activity and Thermostability. A Comparative Study Against N-terminal Conjugation
Source: Appl Biochem Biotechnol. 2026 Feb 7;198(4):3037–60. doi: 10.1007/s12010-025-05545-1 (PMC13032998; doi:10.1007/s12010-025-05545-1)
Supplement: Supplementary file 1 — Supplementary file1 (DOCX 179 KB) [file 12010_2025_5545_MOESM1_ESM.docx]

Cysteine pegylation of a mutant L-asparaginase affords enhanced activity and thermostability. A comparative study against N-terminal conjugation.

## *Rafael B. Ferraro^1,2^, Guilherme R. Benevides^1^, Jheniffer Rabelo, Flaviana da Silva Chaves Marianne A. D. Rodrigues^1^, Gustavo Carretero^3^, Gisele Monteiro^1^, Adalberto Pessoa-Junior^1^, Attilio Converti^4^, Steven Lynham^2^,Paul F. Long^2^ and Carlota O. Rangel-Yagui^1*^*

**^1^**Deparment of Biochemical Pharmaceutical Technology, School of Pharmaceutical Sciences, University of São Paulo, São Paulo, Brazil.

^2^Institute of Pharmaceutical Science, Faculty of Life Sciences & Medicine, King's College London, London, United Kingdom.

^3^Institute of Chemistry, University of São Paulo, São Paulo, Brazil.

^4^Universitá Degli Studi di Genova, Genoa, Italy.

^5^The James Black Center, King’s College London, London, United Kingdom

KEYWORDS: L-Asparaginase, pegylation, enzymatic activity, enzyme stability, thermostability, thermodynamics.


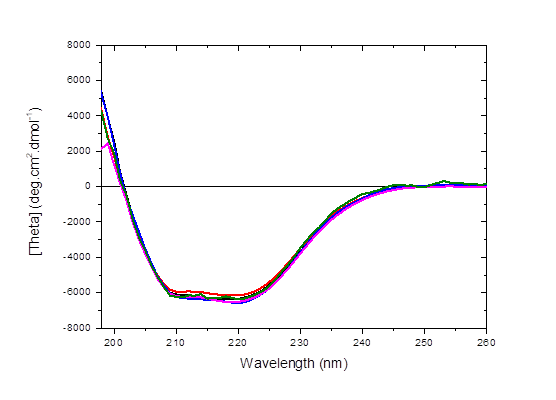


**Figure S1.** Circular Dichroism spectra 200nm – 260 nm wavelenght of mutant ASNase and its pegylated forms. Black line: ASNase; blue line: NT-PEG-ASNase; red line: Cys-PEG-ASNase; pink line: TCEP treated ASNase; green line: poly-PEG-ASNase.

**Chart S1.** Protonation data of ASNase obtained by H++ and Poisson Boltzmann server (PDB2PQR)

| Residue | pKa | | | | | | | |
| --- | --- | --- | --- | --- | --- | --- | --- | --- |
|  | H++ | | | | Poisson Boltzmann Server (PDB2PQR) | | | |
|  | Subunit A | Subunit B | Subunit C | Subunit D | Subunit A | Subunit B | Subunit C | Subunit D |
| N-terminal | 7.2 | 7.3 | 7.6 | 7.8 | --- | --- | --- | --- |
| Lys 22 | >12.0 | >12.0 | >12.0 | >12.0 | 10.6 | 10.2 | 10.6 | 11.4 |
| Lys 28 | >12.0 | >12.0 | >12.0 | >12.0 | 10.4 | 10.3 | 10.3 | 10.4 |
| Lys 43 | >12.0 | >12.0 | >12.0 | >12.0 | 10.4 | 10.6 | 10.4 | 10.3 |
| Lys 49 | >12.0 | >12.0 | >12.0 | >12.0 | 10.4 | 10.5 | 11.1 | 10.2 |
| Lys 71 | >12.0 | >12.0 | >12.0 | >12.0 | 10.0 | 10.2 | 10.0 | 10.2 |
| Lys 72 | >12.0 | >12.0 | >12.0 | >12.0 | 12.0 | 12.3 | 12.2 | 12.2 |
| Lys 79 | >12.0 | >12.0 | >12.0 | >12.0 | 10.8 | 11.4 | 11.2 | 11.3 |
| Lys 104 | >12.0 | >12.0 | >12.0 | >12.0 | 8.7 | 8.7 | 8.9 | 8.8 |
| Lys 107 | >12.0 | >12.0 | >12.0 | >12.0 | 10.2 | 10.3 | 10.2 | 10.1 |
| Lys 139 | >12.0 | >12.0 | >12.0 | >12.0 | 10.5 | 10.5 | 10.5 | 10.5 |
| Lys 162 | >12.0 | >12.0 | >12.0 | >12.0 | 9.9 | 9.9 | 10.0 | 9.8 |
| Lys 172 | >12.0 | >12.0 | >12.0 | >12.0 | 10.2 | 9.0 | 9.0 | 10.3 |
| Lys 186 | >12.0 | >12.0 | >12.0 | >12.0 | 10.9 | 11.0 | 10.2 | 11.0 |
| Lys 196 | >12.0 | >12.0 | >12.0 | >12.0 | 11.2 | 10.2 | 10.5 | 10.5 |
| Lys 207 | >12.0 | >12.0 | >12.0 | >12.0 | 10.6 | 10.6 | 10.6 | 10.6 |
| Lys 213 | >12.0 | >12.0 | >12.0 | >12.0 | 10.8 | 10.8 | 10.7 | 10.9 |
| Lys 229 | >12.0 | >12.0 | >12.0 | >12.0 | 10.8 | 11.2 | 11.5 | 11.5 |
| Lys 251 | >12.0 | >12.0 | >12.0 | >12.0 | 10.5 | 11.2 | 10.5 | 9.7 |
| Lys 262 | >12.0 | >12.0 | >12.0 | >12.0 | 10.4 | 10.4 | 10.3 | 9.7 |
| Lys 288 | >12.0 | >12.0 | >12.0 | >12.0 | 10.8 | 10.9 | 10.8 | 10.8 |
| Lys 301 | >12.0 | >12.0 | >12.0 | >12.0 | 8.3 | 8.4 | 8.4 | 8.5 |
| Lys 314 | >12.0 | >12.0 | >12.0 | >12.0 | 10.5 | 10.5 | 9.6 | 10.4 |

**
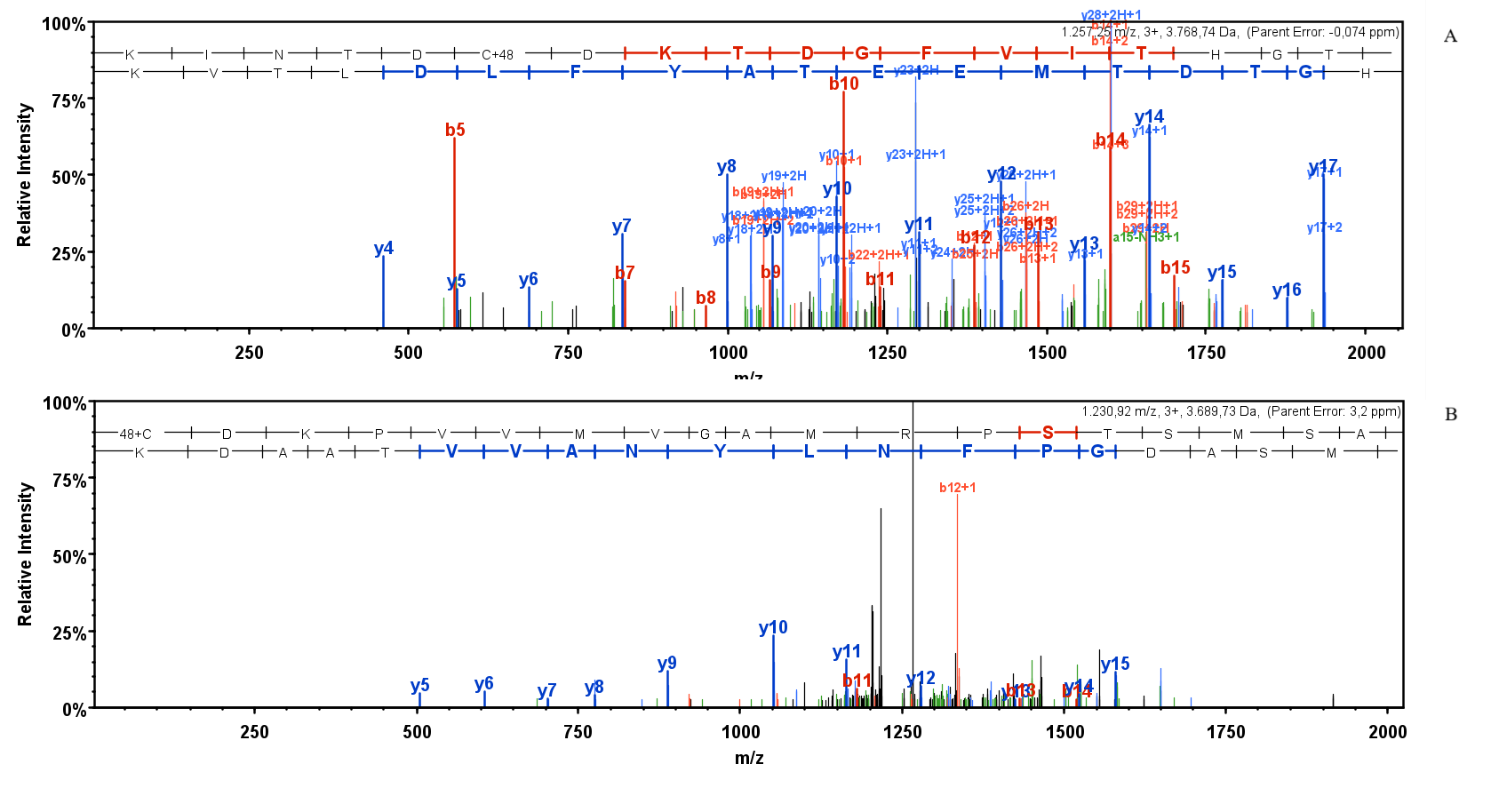
Figure S2:** (A) Fragmentation spectra evidence for a database showing no cysteine oxidation on the peptide ^196^KINTDC_triox_DKTDGFVITHGTDTMEETAYFLDLTVK^207^ with m/z 1527.25^3+^. Supporting Cys77 as unpegylated. (B) Fragmentation spectra evidence for a database assigned trioxidation modification on the peptide ^105^C_triox_DKPVVMVGAMRPSTSMSADGPFNLYNAVVTAADK^139^ with m/z 1230,92^3+^. Supporting Cys105 as unpegylated.
